# A Method for Fuzzy Soft Sets in Decision Making Based on Grey Relational Analysis and D-S Theory of Evidence: Application to Medical Diagnosis

**DOI:** 10.1155/2014/581316

**Published:** 2014-05-19

**Authors:** Ningxin Xie, Guoqiu Wen, Zhaowen Li

**Affiliations:** ^1^College of Information Science and Engineering, Guangxi University for Nationalities, Nanning, Guangxi 530006, China; ^2^College of Science, Guangxi University for Nationalities, Nanning, Guangxi 530006, China

## Abstract

A method based on grey relational analysis and D-S theory of evidence is proposed for fuzzy soft sets in decision making. Firstly, grey relational analysis is used to calculate grey mean relational degrees and determine uncertain degrees of parameters. Then based on uncertain degrees, suitable mass functions of different independent alternatives with different parameters can be constructed. Next, D-S rule of evidence combination is applied to aggregate these alternatives into a collective alternative. Finally, these alternatives are ranked and the best alternative(s) are obtained. Moreover, the effectiveness and feasibility of this method are demonstrated by comparing with the mean potentiality approach and giving an application to medical diagnosis.

## 1. Introduction

There are various types of uncertainty, imprecision, and vagueness in our real life. We do not always successfully deal with the complicated problems with uncertainty by existing theories, such as probability theory, fuzzy set theory [1], and rough set theory [2], which have difficulties as pointed out in [3]. One major problem shared by those theories is their incompatibility with the parameterizations tools.

Molodtsov [3] initiated soft set theory as a new mathematical tool for dealing with uncertainties which classical mathematical tools cannot handle. Recently, there has been a rapid growth of interest in soft set theory. Many efforts have been devoted to further generalizations and extensions of Molodtsov's soft sets. Maji et al. [4] defined fuzzy soft sets by combining soft sets with fuzzy sets; in other words, a degree is attached with the parameterization of fuzzy sets while defining a fuzzy soft set. The study of hybrid models combining soft sets or fuzzy soft sets with other mathematical structures and new operations is emerging as an active research topic of soft set theory [5–7]. Aktaş and Ça$\overset{ˇ}{\text{g}}$man [8] initiated soft groups. Jun applied soft sets to the theory of BCK/BCI algebras and discussed applications of soft sets in ideal theory of BCK/BCI algebras [9–11]. Later Feng et al. [5] defined soft semirings and established a connection between soft sets and semirings. Jiang et al. [6] extended soft sets with description logics. Recently, Feng and Li [12] ascertained the relationships among five different types of soft subsets and considered the free soft algebras associated with soft product operations. It has been shown that soft sets have some nonclassical algebraic properties which are distinct from those of crisp sets or fuzzy sets.

At the same time, there has been some progress concerning practical application of soft set theory, especially the use of soft sets in decision making. Since there is no limitation for the description of the unreal objects in soft sets, researchers can select the form of parameters they require, which immensely simplifies the decision making process and makes it more efficient in the absence of partial information. Maji and Roy [13] first applied soft sets to solve the decision making problem with the help rough approach. Chen [14] defined the parameterization reduction of soft set and discussed its application of decision making problem. Çağman and Enginoğlu [15, 16] investigated soft matrix theory and uni-int decision making, which selected a set of optimum elements from different alternatives. Feng et al. [17] improve and further extend Çağman and Enginoğlu's uni-int decision making method in virtue of choice value soft sets and* k*-satisfaction relations. Moreover, Roy and Maji [18] discussed score value as the evaluation basis to finding an optimal choice object in fuzzy soft sets. But Kong et al. [19] argued that Roy's method was incorrect in general and they proposed a revised algorithm. To address the divergence of different opinions, Feng et al. introduced level soft sets and initiated an adjustable decision making scheme using fuzzy soft sets [20]. Jiang et al. [21] generalized the adjustable approach to fuzzy soft sets based decision making and presented an adjustable approach to intuitionistic fuzzy soft sets based decision making by using level soft sets of intuitionistic fuzzy soft sets. Based on Feng' works, Basu et al. [22] further investigated the fuzzy soft set based decision making and introduced a more efficient fuzzy soft set based decision making method, namely, the mean potentiality approach.

The existing approaches have significant contributions to solve fuzzy soft sets in decision making. However, these approaches are mainly built on the level soft set, and the decision makers select any level soft set with much subjectivity and uncertainty [22]. What's more, there does not exist any unique or uniform criterion for the selection, and the same decision problem may obtain different results from using a different evaluation criterion. As a result, it is difficult to judge which result is right, and we do not know which method or level soft sets should be chosen for selecting the optimal choice object. The key to this problem is how to reduce subjectivity and uncertainty when we choose making decisions method. Then it is necessary to pay attention to this issue.

Grey relational analysis initiated by Deng in 1989 [23] is utilized for generalizing estimates under small samples and uncertain conditions, and it can be regarded as an effective method to solve decision making problems [24–26]. D-S theory of evidence is a new important reasoning method under uncertainty, which has an advantage to deal with subjective judgments and to synthesize the uncertainty knowledge [27]. Compared to probability theory, D-S theory of evidence captures more information to support decision making by identifying the uncertain and unknown evidence. It provides a mechanism to derive solutions from various vague evidences without knowing much prior information. Since it is introduced by Dempster [28] and Shafer [29], D-S theory of evidence has become a hot research issue and has been successfully applied into many fields such as intelligent medical diagnosis [30], knowledge reduction [31], fault diagnosis [32], multiclass classification [33], and supplier selection [34]. Moreover, applying both theories enables the ultimate decision makers to take advantage of both methods' merits and make evaluation experts to deal with uncertainty and risk confidently [34, 35]. The hybrid model has been proved to have its usefulness and versatility in successfully solving a variety of problems in the information sciences, such as data mining, knowledge discovery, and decision making. Therefore, it is very meaningful to explore fuzzy soft set based decision making by using grey relational analysis and D-S theory of evidence.

In the paper, we propose a method for fuzzy soft sets in decision making based on grey relational analysis and D-S theory of evidence and compare the newly proposed method with the mean potentiality approach to fuzzy soft set based decision making. Moreover, we give an illustrative example to interpret the basic principle and an application to medical diagnosis.

The remaining part of this paper is organized as follows. In Section 2, we present some concepts about the soft set, fuzzy soft sets, and D-S theory of evidence. In Section 3, we recall the mean potentiality approach to fuzzy soft sets in decision making and give an example to illustrate this method. In Section 4, we apply grey relational analysis to determine uncertain degrees of parameters and by means of them suitable mass functions with respect to each parameter are constructed. And we use D-S rule of evidence combination to make the decision. In Section 5, the feasibility of this method is demonstrated by comparing with the mean potentiality approach and giving an application to medical diagnosis problems. In Section 6, we conclude this paper.

## 2. Preliminaries

Throughout this paper, *U* denotes an initial universe, *E* denotes the set of all possible parameters, 2^*U*^ denotes the set of all subsets of *U*, and *I* ^*U*^ denotes the set of all fuzzy sets in *U*. We only consider the case where *U* and *E* are both nonempty finite sets.

In this section, we briefly recall some basic concepts about soft set, fuzzy soft sets, the measure of performance of a method, and D-S theory of evidence.

### 2.1. Fuzzy Soft Sets

**Definition (see [3])**

Let *A*⊆*E*. A pair (*F*, *A*) is called a soft set over *U*, if *F* is a mapping defined by *F* : *A* → 2^*U*^.

In other words, a soft set over *U* is a parameterized family of subsets of the universe *U*. For *e* ∈ *A*, *F*(*e*) may be considered as the set of *e*-approximate elements of (*F*, *A*).

To illustrate this idea, let us consider the following example.

**Example**

Let *U* = {*h*_1_, *h*_2_, *h*_3_, *h*_4_, *h*_5_, *h*_6_} be a set of houses and let *A* = {*e*_1_, *e*_2_, *e*_3_, *e*_4_}⊆*E* be a set of status of houses where *e*_*j*_  (*j* = 1,2, 3,4) stands for the parameters “cheap,” “beautiful,” “modern,” and “in the green surroundings,” respectively.

Now, we consider the mapping *F* given by “houses(·),” where (·) is to be filled in by one of the parameters *e*_*j*_ ∈ *A*. For instance, *F*(*e*_1_) means “houses (cheap),” and its functional value is the set consisting of all the cheap houses in *U*.

Let *F*(*e*_1_) = {*h*_1_, *h*_2_, *h*_6_}, *F*(*e*_2_) = {*h*_1_, *h*_6_}, *F*(*e*_3_) = {*h*_3_, *h*_5_}, and *F*(*e*_4_) = {*h*_3_, *h*_4_, *h*_6_}. Then the soft set (*F*, *A*) is a parameterized family {*F*(*e*_*i*_) | *i* = 1,…, 4}, which describes the attractiveness of the houses that Mr. X is going to buy. Besides, (*F*, *A*) is also described in Table 1, in which the value = 1 whenever *h*_*i*_ ∈ *F*(*e*_*j*_)  (1 ⩽ *i* ⩽ 6,1 ⩽ *j* ⩽ 4). Otherwise, the value = 0.

**Definition 3 (see [4])**

Let *A*⊆*E*. A pair (*F*, *A*) is called a fuzzy soft set over *U*, where *F* is a mapping given by *F* : *A* → *I* ^*U*^.

It is easy to see that every soft set may be considered as a fuzzy soft set [20]. Let *x* ∈ *U* and *e* ∈ *A*. *F*(*e*) is a fuzzy subset of *U* and it is called fuzzy value set of parameter *e*. If *F*(*e*) is a crisp subset of *U*, then (*F*, *A*) is degenerated to be the standard soft set. Let *F*(*e*)(*x*) denote the degrees of membership that object *x* holds parameter *e*, and then *F*(*e*) can be written as a fuzzy set such that *F*(*e*) = {*x*/*F*(*e*)(*x*) | *x* ∈ *U*}.

**Example**

Let *U* = {*h*_1_, *h*_2_, *h*_3_, *h*_4_, *h*_5_, *h*_6_} and *A* = {*e*_1_, *e*_2_, *e*_3_, *e*_4_}. Let (*F*, *A*) be a fuzzy soft set over *U*, defined as follows:

(1) F(e1)={h11,h21,h30.2,h40.3,h51,h60.7},F(e2)={h11,h20.1,h30.3,h40.2,h50.1,h60.9},F(e3)={h10.1,h20.4,h31,h41,h50,h60.1},F(e4)={h10.1,h20.3,h31,h41,h50,h61}.

Then (*F*, *A*) is described by Table 2.

**Definition (see [4])**

Let (*F*, *A*) and (*G*, *B*) be two fuzzy soft sets over a common universe *U*. (*F*, *A*) is a fuzzy soft subset of (*G*, *B*) if

- *A*⊆*B*,
- *F*(*e*) is a fuzzy subset of *G*(*e*) for any *e* ∈ *A*.

We write $\left(F,A)\;\tilde{\subset }\;(G,B\right)$.

(*F*, *A*) is said to be a fuzzy soft super set of (*G*, *B*), if (*G*, *B*) is a fuzzy soft subset of (*F*, *A*). We denote it by $\left(F,A)\tilde{⊃}(G,B\right)$.

It is obvious to see that (*F*, *A*) = (*G*, *B*) if and only if $\left(F,A)\tilde{\subset }(G,B\right)$ and $\left(F,A)\tilde{⊃}(G,B\right)$.

**Definition (see [4])**

Let   (*F*, *A*) and (*G*, *B*) be two fuzzy soft sets. Then “(*F*, *A*) AND (*G*, *B*)" is a fuzzy soft set denoted by (*F*, *A*)∧(*G*, *B*) and is defined by (*F*, *A*)∧(*G*, *B*) = (*H*, *A* × *B*), where $H(\alpha ,\beta )=F(\alpha )\tilde{\cap }G(\beta )$ for *α* ∈ *A* and *β* ∈ *B*, where $\tilde{\cap }$ is the operation “fuzzy intersection” of two fuzzy sets.

**Definition (see [22])**

The measure of performance of a method (*M*) which satisfies the optimality criteria to solve a fuzzy soft set in decision making is defined as follows:

(2) ΥM=1∑i=1m∑j=1,i≠jm|F(ei)(Op)−F(ej)(Op)|+∑i=1mF(ei)(Op),

where *m* is the number of choice parameters and *F*(*e*_*i*_)(*O*_*p*_) is the membership value of the optimal object *O*_*p*_ for the choice parameter *e*_*i*_.

Suppose there are two methods *M*_1_, *M*_2_ which satisfy the optimality criteria and their measures of performances are, respectively, *Υ*_*M*_1__ and *Υ*_*M*_2__. If *Υ*_*M*_1__ > *Υ*_*M*_2__, then *M*_1_ is better than *M*_2_. If *Υ*_*M*_1__ < *Υ*_*M*_2__, then *M*_2_ is better than *M*_1_. If *Υ*_*M*_1__ = *Υ*_*M*_2__, then the performances of the both methods are the same.

### 2.2. Basic Concepts of D-S Theory of Evidence

D-S theory of evidence is an important reasoning method under uncertainty. It has an advantage to deal with subjective judgments and to synthesize the uncertainty knowledge [27]. D-S theory of evidence discusses a frame of discernment, denoted by Θ, which is a finite nonempty set of mutually exclusive and exhaustive hypotheses (or all possible outcomes of an event), denoted by {*A*_1_, *A*_2_,…, *A*_*n*_} and *A*_*i*_∩*A*_*j*_ = *∅*. 2^Θ^ denotes the set of all subsets of Θ.

**Definition (see [29])**

Let Θ be a frame of discernment. A basic probability assignment function (for short mass function) on Θ is defined a mapping *m* : 2^Θ^ → [0,1], *m* satisfies

(3) $$\begin{array}{c} m\left(\emptyset \right)=0,\;\;\underset{A\subseteq \Theta }{\sum }m\left(A\right)=1. \end{array}$$

For any *A*⊆Θ, *m*(*A*) represents the belief measure that one is willing to commit exactly to *A*, given a certain piece of evidence.

**Definition (see [29])**

Let Θ be the frame of discernment and let *m* : 2^Θ^ → [0,1] be a mass function. Then a belief function on Θ is defined as a mapping Bel : 2^Θ^ → [0,1], and Bel satisfies

(4) $$\begin{array}{c} \text{Bel}\left(\emptyset \right)=0,\;\;\text{Bel}\left(\Theta \right)=1,\\ \text{Bel}\left(A\right)=\underset{B\subseteq A}{\sum }m\left(B\right),\;\forall A\subseteq \Theta . \end{array}$$

Bel(*A*) represents the sum of possibilities measurements of all subsets of *A*, namely, the total degree of support of *A*. Belief function represents the imprecision and uncertainty in the decision making process. In the case of singleton element, Bel(*A*) = *m*(*A*).

In reality, a decision maker can often gain access to more than one information source in order to make his/her decisions. The evidence theory constructs a set of hypotheses of known mass function values from these information sources and then computes a new set of combined evidences. This construction rule is called D-S rule of evidence combination for group aggregation.

**Definition (see [29])**

Let Θ be the frame of discernment. Suppose there are two mass functions: *m*_1_ and *m*_2_ over Θ, induced by two independent items of evidences *A*_1_, *A*_2_,…, *A*_*s*_ and *B*_1_, *B*_2_,…, *B*_*t*_, respectively. D-S rule of evidence combination is as follows:

(5) m(A)=m1⊕m2(A)={11−K∑Ai∩Bj=Am1(Ai)m2(Bj),∀A⊆Θ,  A≠∅,0,A=∅,

where *K* = ∑_*A*_*i*_∩*B*_*j*_=*∅*_ *m*_1_(*A*_*i*_)*m*_2_(*B*_*j*_) < 1. *K* is called the conflict probability and reflects the extent of the conflict between the evidences. Coefficient 1/(1 − *K*) is called normalized factor, and its role is to avoid the probability of assigning non-0 to empty set *∅* in the combination.

The synthesis of multiple evidences can be promoted according to D-S rule of evidence combination:

(6) m1⊕m2⋯⊕mn(A) =11−K∑⋂i=1nAi=A,Ai⊆Θm1(A1)m2(A2)⋯mn(An),

where *A*⊆Θ, *A* ≠ *∅*, and *K* = ∑_⋂_*i*=1_^*n*^*A*_*i*_=*∅*,*A*_*i*_⊆Θ_ *m*_1_(*A*_1_)*m*_2_(*A*_2_) ⋯ *m*_*n*_(*A*_*n*_) < 1.

D-S rule of evidence combination can increase belief measure and reduce the uncertain degree of the whole evidences to improve reliability.

**Example**

Let Θ = {*A*_1_, *A*_2_} be the frame of discernment. Suppose there are two mass functions *m*_1_ and *m*_2_ over Θ, induced by an independent piece of evidences *A*_1_, *A*_2_, given by

(7) $$\begin{array}{c} m_{1}\left(A_{1}\right)=0.2,\;\;m_{1}\left(A_{2}\right)=0.5,\;\;m_{1}\left(\Theta \right)=0.3,\\ m_{2}\left(A_{1}\right)=0.4,\;\;m_{2}\left(A_{2}\right)=0.3,\;\;m_{2}\left(\Theta \right)=0.3. \end{array}$$

We apply D-S rule of evidence combination to combine the two evidences and then have

(8) m(A1) =m1⊕m2(A1) =m1(A1)m2(A1)+m1(A1)m2(Θ)+m1(Θ)m2(A1)1−K =0.3514,m(A2) =m1⊕m2(A2) =m1(A2)m2(A2)+m1(A2)m2(Θ)+m1(Θ)m2(A2)1−K =0.5270,m(Θ)=m1⊕m2(Θ)=m1(Θ)m2(Θ)1−K=0.1216,

where *K* = *m*_1_(*A*_1_)*m*_2_(*A*_2_) + *m*_1_(*A*_2_)*m*_2_(*A*_1_) = 0.26.

## 3. Mean Potentiality Approach

Like most of decision making problems, fuzzy soft sets based on decision making involve the evaluation of all decision alternatives. Recently, applications of fuzzy soft set based on decision making have attracted more and more attentions. The works of Roy et al. [18–20] are fundamental and significant. Later Kong et al. [36] applied grey relational analysis to solve fuzzy soft sets in decision making. Generally, there does not exist any unique or uniform criterion for the evaluation of decision alternatives under uncertain conditions. Thus, Basu et al. [22] further studied and proposed the mean potentiality approach to fuzzy soft sets in decision making, which is more deterministic and accurate than Feng's approach [20].

### 3.1. Basu's Approach

Let *U* = {*x*_1_, *x*_2_,…, *x*_*m*_} be a finite universe set and let *A* = {*e*_1_, *e*_2_,…, *e*_*n*_} be a choice parameter set. Given a fuzzy soft set (*F*, *A*), *F*(*e*_*j*_)(*x*_*i*_) denotes the membership value that object *x*_*i*_ holds parameter *e*_*j*_. *ρ* denotes the maximum number of significant figures among all the membership values of the objects concerned with (*F*, *A*). Next, we mainly recall the mean potentiality approach to (*F*, *A*) based on decision making problem with equally weighted choice parameters.

**Step 1**

Find a normal parameter reduction *B* of *A*. If it exists, we construct the tabular representation of (*F*, *B*). Otherwise, we construct the tabular representation of (*F*, *A*) with the choice values of each object.

**Step 2**

Compute the mean potentiality *m*_*p*_ = ∑_*i*=1_^*m*^∑_*j*=1_^*n*^ *F*(*e*_*j*_)(*x*_*i*_)/(*m* × *n*) up to *ρ* significant figures, denoted by *m*_*p*_′.

**Step 3**

Construct a *m*_*p*_′-level soft set of (*F*, *A*), represent it in tabular form, and then compute the choice value *c*_*i*_ for each *x*_*i*_.

**Step 4**

Denote *max*{*c*_1_, *c*_2_,…, *c*_*m*_} = *c*_*k*_. If *c*_*k*_ is unique, then the optimal choice object is *x*_*k*_ and the process will be stopped. Otherwise, go to Step 5.

**Step 5**

Compute the nonnegative difference between the largest and the smallest membership value in each column (resp., each row) and denote it as *a*_*j*_(*j* = 1,2,…, *n*) (resp., *β*_*i*_(*i* = 1,2,…, *m*)).

**Step 6**

Compute the average *α* = (∑_*j*=1_^*n*^ *a*_*j*_)/*n* up to *ρ* significant figures, denoted by *α*′.

**Step 7**

Construct a *α*′-level soft set of (*F*, *A*), represent it in tabular form, and then compute the choice value *c*_*i*_′ for each *x*_*i*_.

**Step 8**

Denote *max*{*c*_1_′, *c*_2_′,…, *c*_*m*_′} = *c*_*l*_′. If *c*_*l*_′ is unique, then the optimal choice object is *x*_*l*_ and the process will be stopped. Otherwise, go to Step 9.

**Step 9**

Consider the object corresponding to the minimum value of *β*_*i*_(*i* = 1,2,…, *m*) as the optimal choice of decision makers.

### 3.2. Example Illustration

In this subsection, we give the following example to illustrate the mean potentiality approach to fuzzy soft sets in decision making.

**Example**

Let us apply the mean potentiality approach to consider a decision making problem which is associated with the fuzzy soft set (*F*, *A*) given in Table 3.

1. Since *A* is indispensable, there does not exist any normal parameter reduction of *A*.
2. The mean potentiality of (*F*, *A*) is *m*_*p*_ = ∑_*i*=1_^3^∑_*j*=1_^5^ *F*(*e*_*j*_)(*x*_*i*_)/(3 × 5) = 0.626; thus, *m*_*p*_′ = 0.63.
3. *m*_*p*_′-level soft set of (*F*, *A*) with choice values is given by Table 4.
4. Since *x*_1_ and *x*_2_ have the same maximum choice values (3), we have to calculate the *α*_*j*_ and *β*_*i*_ values of (*F*, *A*).
5. See Table 5
6. Now *α* = (0.29 + 0.31 + 0.44 + 0.32 + 0.32)/5 = 0.336; thus, *α*′ = 0.34.
7. So the *α*′-level soft set of (*F*, *A*) with choice values *c*_*i*_′  (*i* = 1,2, 3) is given by Table 6.
8. Here max{*c*_1_′, *c*_2_′, *c*_3_′} = {*c*_2_′, *c*_3_′}, that is, not unique, we have to consider the *β*_2_ and *β*_3_.
9. Since min{*β*_2_, *β*_3_} = *β*_3_( = 0.37), *x*_3_ is the optimal choice object.

## 4. A Method for Fuzzy Soft Sets in Decision Making Based on Grey Relational Analysis and D-S Theory of Evidence

The existing approaches to fuzzy soft sets in decision making are mainly based on the level soft set to obtain useful information such as choice values and score values. However, it is very difficult for decision makers to select a suitable level soft set. Inspired by the work of Wu [34] and Li and Liu[35], we introduce a method for fuzzy soft sets in decision making based on grey relational analysis and D-S theory of evidence. It not only allows us to avoid the problem of selecting the suitable level soft set but also helps reducing uncertainty caused by people's subjective cognition so as to raise the choice decision level.

This method may include three phases. First, grey relational analysis is applied to calculate the grey mean relational degree between each independent alternative and the mean of all alternatives with each parameter, and the uncertain degree of each parameter is obtained. Second, the suitable mass function with respect to each parameter (or evidence) is constructed by the uncertain degree of each parameter. Third, we apply D-S rule of evidence combination to aggregate independent evidences into a collective evidence, by which the candidate alternatives are ranked and the best alternative(s) are obtained.

### 4.1. Method Illustration

In the following, we consider a decision making problem concerned with *m* mutually exclusive alternatives *x*_*i*_ and *n* evaluation parameters (or indexes) *e*_*j*_. *d*_*ij*_ denotes the membership value of *x*_*i*_ with *e*_*j*_. Put

(9) $$\begin{array}{c} \Theta =\left\{x_{1},x_{2},\ldots ,x_{m}\right\},\;\;A=\left\{e_{1},e_{2},\ldots ,e_{n}\right\}. \end{array}$$

Define *F* : *A* → *I* ^Θ^ by *F*(*e*_*j*_)(*x*_*i*_) = *d*_*ij*_. Then (*F*, *A*) is a fuzzy soft set over Θ and *D* = (*d*_*ij*_)_*m*×*n*_ is called a fuzzy soft decision matrix induced by (*F*, *A*).

In this paper, we can consider the parameter set of the decision making as a set of evidence.

Compared to probability theory, D-S theory of evidence captures more information to support decision making, by identifying the uncertain and unknown evidences. It provides a mechanism to derive solutions from various vague evidences (parameter) without knowing much prior information. We must get mass functions of alternatives with each evidence (parameter) beforehand if we apply D-S theory of evidence to make decisions. However, how to find uncertain degree of the evidence (parameter) is a critical problem. Grey relational analysis is employed as a means to reflect uncertainty between experts in multiple parameter models through the membership value. Next, we apply grey relational analysis to obtain uncertain degree of the evidence (parameter).

Firstly, we present some basic notions.

**Definition (see [37])**

Let Θ = {*x*_1_, *x*_2_,…, *x*_*m*_} and *A* = {*e*_1_, *e*_2_,…, *e*_*n*_} and let (*F*, *A*) be a fuzzy soft set on Θ. Suppose that *D* = (*d*_*ij*_)_*m*×*n*_ is a fuzzy soft decision matrix induced by (*F*, *A*). For any *i*, *j*, denote

(10) $$\begin{array}{c} \tilde{d_{i}}=\frac{1}{n}\overset{n}{\underset{j=1}{\sum }}d_{ij},\;\;\Delta d_{ij}=\left|d_{ij}-\tilde{d_{i}}\right|,\\ r_{ij}=\frac{\min_{1⩽i⩽m\;}\Delta d_{ij}+\rho \max_{1⩽i⩽m\;}\Delta d_{ij}}{\Delta d_{ij}+\rho \max_{1⩽i⩽m\;}\Delta d_{ij}},\;\;\text{where}\;\rho \in \left(0,1\right),\\ DOI\left(e_{j}\right)=\frac{1}{m}{\left(\overset{m}{\underset{i=1}{\sum }}{\left(r_{ij}\right)}^{q}\right)}^{1/q}\;\left(j=1,2,\ldots ,n\right). \end{array}$$

Then

1. $\tilde{d_{i}}$ is called the mean of all parameters with respect to *x*_*i*_,
2. Δ*d*_*ij*_ is called the difference information between *d*_*ij*_ and $\tilde{d_{i}}$,
3. *ρ* is called the distinguishing coefficient and *r*_*ij*_ is called the grey mean relational degree between *d*_*ij*_ and $\tilde{d_{i}}$,
4. *DOI*(*e*_*j*_) is called *q* order uncertain degree of the parameter *e*_*j*_.

The purpose of the distinguishing coefficient *ρ* is to expand or compress the range of the grey relational coefficient. In this paper, we pick *ρ* = 0.5, *q* = 2 to obtain strong distinguishing effectiveness.

It is worthy to notice that this method to obtain the uncertain degree in Definition 13 varies from different situations. Since a parameter is specially more matching with the mean of the parameter set than other parameters, the parameter contains more satisfying information for decision making and the uncertain degree of the parameter with respect to alternatives is lower. Then, in this paper we just consider grey mean relational degree between *d*_*ij*_ and $\tilde{d_{i}}$.

**Definition (see [38])**

Let *X* = (*x*_1_, *x*_2_,…, *x*_*m*_) be a finite difference information sequence, where there exists *x*_*i*_*k*__ ≠ 0 for *k* = 1,2,…, *m* and 1 ⩽ *i*_*k*_ ⩽ *m*. Then the information structure image sequence *Y* = (*y*_1_, *y*_2_,…, *y*_*m*_) is given by *y*_*i*_ = *x*_*i*_/∑_*i*=1_^*m*^ *x*_*i*_.

In a fuzzy soft decision matrix *D* = (*d*_*ij*_)_*m*×*n*_ concerned with *m* mutually exclusive alternatives *x*_*i*_ and *n* evaluation parameters *e*_*j*_, where *d*_*ij*_ is the membership value of *x*_*i*_ with *e*_*j*_. The information structure image sequence with respect to *e*_*j*_ is denoted by $d_{j}=\{\tilde{d_{1j}},\tilde{d_{2j}},\tilde{d_{3j}},\ldots ,\tilde{d_{mj}}\}$, where $\tilde{d_{ij}}=d_{ij}/\sum_{i=1}^{m}d_{ij}$. Then we obtain an information structure image metric by *d*_*j*_  (*j* = 1,2,…, *n*).

D-S theory of evidence is a powerful method for combining accumulative evidences of changing prior opinions in the light of new evidences [29]. The primary procedure about combining the known evidences or information with other evidences is to construct suitable mass functions of evidences. It is flexible to obtain mass function, and people's experience, knowledge, or thinking will affect the selection of mass function.

Now, by the uncertain degree of each parameter, we can obtain mass function of each alternative with respect to each parameter.

**Theorem 15**

Let Θ = {*x*_1_, *x*_2_,…, *x*_*m*_} and *A* = {*e*_1_, *e*_2_,…, *e*_*n*_} and let (*F*, *A*) be a fuzzy soft set on Θ. Suppose that *D* = (*d*_*ij*_)_*m*×*n*_ is a fuzzy soft decision matrix induced by (*F*, *A*), where *d*_*ij*_ denotes the membership value that the alternative *x*_*i*_ holds the parameter *e*_*j*_. Denote $\tilde{d_{ij}}=d_{ij}/\sum_{i=1}^{m}d_{ij}$. We define functions *m*_*e*_*j*__  (*j* = 1,2,…, *n*) with respect to the parameter *e*_*j*_, and it satisfies

(11) mej(xi)=dij~(1−DOI(ej))(i=1,2,…,m,  j=1,2,…,n),mej(Θ)=1−∑i=1mmj(i) (j=1,2,…,n).

Then *m*_*e*_*j*__  (*j* = 1,2,…, *n*) are mass functions.

In a fuzzy soft decision matrix *D* = (*d*_*ij*_)_*m*×*n*_, denote *m*_*e*_*j*__(*x*_*i*_), *m*_*e*_*j*__(Θ) by *m*_*j*_(*i*) and *m*_*j*_(*m* + 1), respectively. *m*_*j*_(*i*) implies the belief measure of the alternative *x*_*i*_ with the parameter *e*_*j*_. *m*_*j*_(*m* + 1) implies the belief measure of the whole uncertainty with respect to the parameter *e*_*j*_.

Next, using D-S rule of evidence combination to compose *m*_1_, *m*_2_,…, *m*_*n*_ with respect to each alternative *x*_*i*_, we get the belief measure of each alternative *x*_*i*_. Thus we obtain decision making results.

Based on the above analysis, given a fuzzy soft set (*F*, *E*) concerned with *m* mutually exclusive alternatives *x*_*i*_ and *n* evaluation parameters *e*_*j*_, the decision procedure of the proposed method for (*F*, *E*) can be summarized as follows.

**Step 1**

Construct a fuzzy soft decision matrix *D* = (*d*_*ij*_)_*m*×*n*_ induced by (*F*, *A*), where *d*_*ij*_ is the membership value of *x*_*i*_ with *e*_*j*_.

**Step 2**

Calculate the mean of all parameters with respect to each alternative by

(12) $$\begin{array}{c} \tilde{d_{i}}=\frac{1}{n}\overset{n}{\underset{j=1}{\sum }}d_{ij}\;\left(i=1,2,\ldots ,m\right). \end{array}$$

**Step 3**

Calculate the difference information between *d*_*ij*_ and $\tilde{d_{i}}$ and construct the difference matrix by

(13) $$\begin{array}{c} \Delta d_{ij}=\left|d_{ij}-\tilde{d_{i}}\right|,\;\Delta D={\left(\Delta d_{ij}\right)}_{m\times n}\\ \left(i=1,2,\ldots ,m,\;\;j=1,2,\ldots ,n\right). \end{array}$$

**Step 4**

Calculate gray mean relational degrees between *d*_*ij*_ and $\tilde{d_{i}}$ by

(14) rij=min⁡1⩽i⩽m Δdij+0.5  max⁡1⩽i⩽m ΔdijΔdij+0.5max⁡1⩽i⩽m Δdij(i=1,2,…,m, j=1,2,…,n).

**Step 5**

Calculate the uncertain degree of each parameter *e*_*j*_ by

(15) $$\begin{array}{c} DOI\left(e_{j}\right)=\frac{1}{m}{\left(\overset{m}{\underset{i=1}{\sum }}{\left(r_{ij}\right)}^{2}\right)}^{1/2}\;\left(j=1,2,\ldots ,n\right). \end{array}$$

**Step 6**

Calculate the information structure image sequence *d*_*j*_ with respect to each parameter *e*_*j*_ and construct the matrix by Definition 14.

**Step 7**

Calculate mass function values of each alternative *x*_*i*_ and Θ with respect to each parameter *e*_*j*_ by Theorem 15.

**Step 8**

Calculate belief measure of each alternative *x*_*i*_ by Definition 10.

**Step 9**

Obtain decision making. The decision is *x*_*k*_ if *c*_*k*_ = max⁡⁡Bel({*x*_*i*_}). Optimal choices have more than one object if there are more alternatives corresponding to the maximum.

### 4.2. Example Illustration

In this subsection, we give the following example to illustrate the newly proposed method for fuzzy soft sets in decision making.

**Example**

Using the newly proposed method, we reconsider the fuzzy soft set (*F*, *A*) given in Example 12.

Now, we suppose that the three mutually exclusive and exhaustive alternatives construct a frame of discernment, denoted by Θ = {*x*_1_, *x*_2_, *x*_3_}. And we consider the set of parameters *A* = {*e*_1_, *e*_2_, *e*_3_, *e*_4_, *e*_5_} as a set of evidences.

(1) Construct a fuzzy soft decision matrix induced by (*F*, *A*) as follows:
  (16) $$\begin{array}{c} D={\left(d_{ij}\right)}_{3\times 5}=\left(\begin{array}{ccccc} 0.85 & 0.71 & 0.38 & 0.32 & 0.75\\ 0.56 & 0.82 & 0.76 & 0.64 & 0.43\\ 0.84 & 0.51 & 0.82 & 0.53 & 0.47 \end{array}\right). \end{array}$$
(2) Calculate the mean of all parameters of each alternative *x*_*i*_ as follows:
  (17) $$\begin{array}{c} \tilde{x_{1}}=0.6020,\;\;\tilde{x_{2}}=0.6420,\;\;\tilde{x_{3}}=0.6340. \end{array}$$
(3) Calculate the difference information between *d*_*ij*_ and $\tilde{x_{i}}$, and construct the difference matrix as follows:
  (18) $$\begin{array}{c} \Delta D=\left(\begin{array}{ccccc} 0.2480 & 0.1080 & 0.2220 & 0.2820 & 0.1480\\ 0.0820 & 0.1780 & 0.1180 & 0.0020 & 0.2120\\ 0.2060 & 0.1240 & 0.1860 & 0.1040 & 0.1640 \end{array}\right). \end{array}$$
(4) Calculate the gray mean relational degree between *d*_*ij*_ and $\tilde{x_{i}}$ based on Δ*D* as follows:
  (19) $$\begin{array}{c} {\left(r_{ij}\right)}_{3\times 5}=\left(\begin{array}{ccccc} 0.5538 & 1.0000 & 0.6877 & 0.3381 & 1.0000\\ 1.0000 & 0.7378 & 1.0000 & 1.0000 & 0.7987\\ 0.6242 & 0.9249 & 0.7710 & 0.5837 & 0.9407 \end{array}\right). \end{array}$$

(5) Calculate the uncertain degree of each parameter *e*_*j*_ as follows:
  (20) $$\begin{array}{c} DOI\left(e_{1}\right)=0.4341,\;\;DOI\left(e_{2}\right)=0.5164,\\ DOI\left(e_{3}\right)=0.4793,\;\;DOI\left(e_{4}\right)=0.4021,\\ DOI\left(e_{5}\right)=0.5295. \end{array}$$
(6) Calculate the information structure image sequence with respect to each parameter *e*_*j*_ and construct the matrix as follows:
  (21) $$\begin{array}{c} \tilde{D}={\left(\tilde{d_{ij}}\right)}_{3\times 5}=\left(\begin{array}{ccccc} 0.3778 & 0.3480 & 0.1939 & 0.2148 & 0.4545\\ 0.2489 & 0.4020 & 0.3878 & 0.4295 & 0.2606\\ 0.3733 & 0.2500 & 0.4184 & 0.3557 & 0.2848 \end{array}\right). \end{array}$$
(7) For any *A* ∈ 2^Θ^ with |*A* | = 0 or 2, put *m*(*A*) = 0. We calculate mass function values of each alternative *x*_*i*_ and Θ with respect to parameter *e*_*j*_ by Theorem 15:
  (22) $$\begin{array}{c} {\left(m_{j}\left(i\right)\right)}_{3\times 5}=\left(\begin{array}{ccccc} 0.2138 & 0.1683 & 0.1010 & 0.1284 & 0.2139\\ 0.1408 & 0.1944 & 0.2019 & 0.2568 & 0.1226\\ 0.2113 & 0.1209 & 0.2179 & 0.2127 & 0.1340 \end{array}\right),\\ m_{1}\left(4\right)=0.4341,\;\;m_{2}\left(4\right)=0.5164,\;\;m_{3}\left(4\right)=0.4793,\\ m_{4}\left(4\right)=0.4021,\;\;m_{5}\left(4\right)=0.5295. \end{array}$$
 The mean of belief measure of the whole uncertainty is (0.4341 + 0.5164 + 0.4793 + 0.4021 + 0.5295)/5 = 0.4723.
(8) Calculate belief measure of each alternative *x*_*i*_ by combining these evidences, respectively, by Definition 7:
  (23) $$\begin{array}{c} \text{Bel}\left(\left\{x_{1}\right\}\right)=m_{1}⊕m_{2}⊕m_{3}\cdots ⊕m_{5}\left(\left\{x_{1}\right\}\right)=0.2690,\\ \text{Bel}\left(\left\{x_{2}\right\}\right)=m_{1}⊕m_{2}⊕m_{3}\cdots ⊕m_{5}\left(\left\{x_{2}\right\}\right)=0.3309,\\ \text{Bel}\left(\left\{x_{3}\right\}\right)=m_{1}⊕m_{2}⊕m_{3}\cdots ⊕m_{5}\left(\left\{x_{3}\right\}\right)=0.3218,\\ \text{Bel}\left(\Theta \right)=m_{1}⊕m_{2}⊕m_{3}\cdots ⊕m_{5}\left(\Theta \right)=0.0782. \end{array}$$
 Then the final rang order is *x*_2_≻*x*_3_≻*x*_1_.
(9) According to the maximum belief measure principle, the optimally choice decision is *x*_2_, which is different from the choice decision by using the mean potentiality approach in Example 12.

Now, by Definition 7, we can calculate the measure of performance of our method *Υ* = 3.7202 and the measure of performance of the mean potentiality approach *Υ* = 3.6462.

From the above results, the belief measure of the whole uncertainty falls from the initial mean 0.4723 to 0.0782 after evidence combination. This implies that our method that combined grey relational analysis with D-S theory of evidence can help reducing uncertainty caused by people's subjective cognition so as to raise the choice decision level. Moreover, judged by the measures *Υ* of performance, our method is more accurate and effective than the mean potentiality approach under uncertain information.

## 5. An Application to Medical Diagnosis

A major task of medical science is to diagnose diseases. Generally a patient suffering from a disease may have multiple symptoms and the information available to physician about his patient is inherently uncertain. And it is also observed that there are certain symptoms which may be common to more than one disease leading to diagnostic dilemma. Doctors always detect clinical manifestations by the comparison with predefined classes to find the most similar disease. Only one comprehensive result can be gotten from existing methods for medical diagnosis, which cannot provide the certainty or uncertainty of the result. One of the toughest challenges in medical diagnosis is handling uncertainty. Therefore, it is necessary to find another method to deal with the unknown factors in the process of medical diagnosis and improve level of medical diagnosis, and then we apply the above proposed method to solve this medical diagnosis problem.

Now we consider a medical diagnosis problem with seven symptoms such as fever, running nose, weakness, orofacial pain, nausea vomiting, swelling, and trismus which have more or less contribution in four diseases such as acute dental abscess, migraine, acute sinusitis, and peritonsillar abscess. Now, from medical statistics, the degree of availability of these seven symptoms in these four diseases is observed as follows. The belonging degrees of these four diseases with seven symptoms “fever”, “running nose”, “weakness”, “orofacial pain”, “nausea vomiting”, “swelling” and “trismus”, respectively are {0.6,0, 0.6,0.9,0, 0.7,0.8}, {0.2,0, 0.1,0.9,0.8,0, 0}, {0.3,0.7,0.3,0.8,0.3,0.4,0} and {0.4,0, 0.2,0.7,0.1,0.6,0.5}. The belonging degrees of these four diseases with three detecting tools “history”, “physical examination” and “laboratory investigation” are {0.6,0.8,0.4}, {0.8,0.3,0.6}, {0.8,0.4,0.7} and {0.6,0.8,0.3}, respectively. Suppose a patient who is suffering a disease has the symptoms: fever, running nose, and orofacial pain and is diagnosed by the three tools. Now the problem is how a doctor detects the actual disease with effective symptoms and diagnosed tools among these four diseases for that patient. To solve this problem, we first detect the disease which is most suited with the observed symptoms of the patient and then we find the actual symptoms which are optimal for that disease. These can be solved by using the above proposed method. For solving these the following notations are used:

- {fever, running nose, weakness, orofacial pain, nausea vomiting, swelling, trismus, history, physical examination, and laboratory investigation} = {*e*_1_, *e*_2_, *e*_3_, *e*_4_, *e*_5_, *e*_6_, *e*_7_, *s*_1_, *s*_2_, *s*_3_},
- {acute dental abscess, migraine, acute sinusitis, and peritonsillar abscess} = {*d*_1_, *d*_2_, *d*_3_, *d*_4_}

Therefore, in the parlance of fuzzy soft set, the finite universe, *U* = {*d*_1_, *d*_2_, *d*_3_, *d*_4_} and the set of parameters *E* = {*e*_1_, *e*_2_, *e*_3_, *e*_4_, *e*_5_, *e*_6_, *e*_7_, *s*_1_, *s*_2_, *s*_3_}, *A* = {*e*_1_, *e*_2_, *e*_4_}, *B* = {*s*_1_, *s*_2_, *s*_3_}.

Now the fuzzy soft set (*F*, *A*) is defined as

(24) F(e1)={d10.6,d20.2,d30.3,d40.4},F(e2)={d10,d20,d30.7,d40},F(e4)={d10.9,d20.9,d30.8,d40.7}.

And the fuzzy soft set (*F*, *B*) is defined as

(25) F(s1)={d10.6,d20.8,d30.8,d40.6},F(s2)={d10.8,d20.3,d30.4,d40.8},F(s3)={d10.4,d20.6,d30.7,d40.3}.

The two fuzzy soft sets (*F*, *A*) and (*G*, *B*) are describing “symptoms of the diseases” and “decision making tools of the diseases,” respectively. The tabular representation of (*F*, *A*) and (*G*, *B*) is given in Tables 7 and 8, respectively.

The key problem is how a doctor reaches to the most suitable diagnosis according to these symptoms, history, physical examination, and laboratory investigation of the patient. To solve this problem, we consider “(*F*, *A*) AND (*G*, *B*),” given by Table 9. There are four diseases *d*_1_, *d*_2_, *d*_3_, *d*_4_, and nine pairs of parameters *a*_1_ = (*e*_1_, *s*_1_), *a*_2_ = (*e*_1_, *s*_2_), *a*_3_ = (*e*_1_, *s*_3_), *a*_4_ = (*e*_2_, *s*_1_), *a*_5_ = (*e*_2_, *s*_2_), *a*_6_ = (*e*_2_, *s*_3_), *a*_7_ = (*e*_4_, *s*_1_), *a*_8_ = (*e*_4_, *s*_2_), *a*_9_ = (*e*_4_, *s*_3_), which is a pair of one symptom and one decision making tool, respectively.

Next we will apply our method to detect which disease is most suited with the symptoms and these investigative procedures. Then, in the decision making, we consider that the four diseases construct a frame of discernment, denoted by Θ = {*d*_1_, *d*_2_, *d*_3_, *d*_4_}. We consider the nine pairs of parameters as a set of evidences, which contains a diagnosis parameter system, denoted by *P* = {*a*_1_, *a*_2_, *a*_3_, *a*_4_, *a*_5_, *a*_6_, *a*_7_, *a*_8_, *a*_9_}.

**Step 1**

We construct a fuzzy soft decision matrix induced by “(*F*, *A*) AND (*G*, *B*),” which completely presents the degree that a patient is suffering from a disease *d*_*i*_ with one symptom and one decision making tool *a*_*j*_:

(26) D=(dij)4×9=(0.60.60.40000.60.80.40.20.20.20000.80.30.60.30.30.30.70.40.70.80.40.70.40.40.30000.60.70.3).

**Step 2**

Since *a*_*j*_ is specially more matching the mean of the parameter set than other parameters, *a*_*j*_ contains the satisfying information for decision making and the uncertain degree of *a*_*j*_ is low. Now, we consider the mean $\tilde{d_{i}}$ of the parameter set with respect to *d*_*i*_, calculated by $\tilde{d_{i}}=(1/9)\sum_{j=1}^{9}d_{ij}$ as follows:

(27) $$\begin{array}{c} \tilde{d_{1}}=0.3778,\;\;\tilde{d_{2}}=0.2556,\\ \tilde{d_{3}}=0.5111,\;\;\tilde{d_{4}}=0.3000. \end{array}$$

**Step 3**

To obtain gray mean relational degrees, we need to calculate the difference information between *d*_*ij*_ and $\tilde{d_{i}}$ and construct a difference matrix as follows:

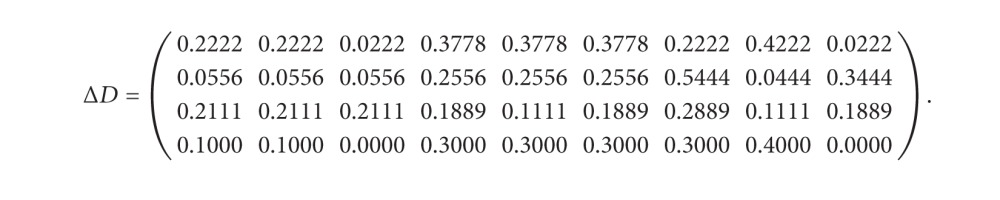
(28)

**Step 4**

Based on Δ*D*, the gray mean relational degree between *d*_*ij*_ and $\tilde{d_{i}}$ is calculated as follows:

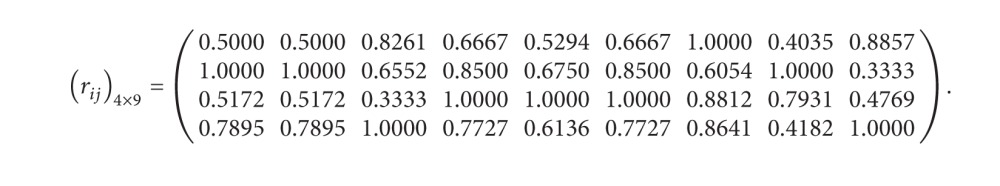
(29)

**Step 5**

In order to obtain mass functions of *d*_*i*_ and Θ with respect to *a*_*j*_, now we need to calculate the uncertain degree of *a*_*j*_ as follows:

(30) DOI(a1)=0.3658,  DOI(a2)=0.3658,DOI(a3)=0.3727,  DOI(a4)=0.4156,DOI(a5)=0.3634,  DOI(a6)=0.4156,DOI(a7)=0.4250,  DOI(a8)=0.3506,DOI(a9)=0.3643.

**Step 6**

We calculate information structure image sequences with respect to each *a*_*j*_ and construct a matrix as follows:

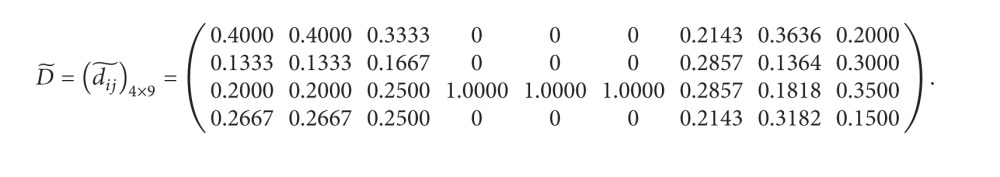
(31)

**Step 7**

For any *A* ∈ 2^Θ^ with |*A* | = 0,2, 3, put *m*(*A*) = 0. We calculate mass function values of *d*_*i*_ and Θ with respect to *a*_*j*_ by Theorem 15 as follows:

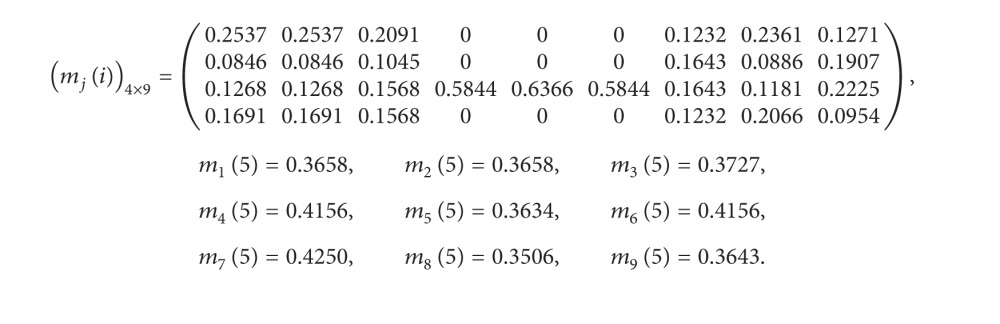
(32)

The mean of belief measure of the whole uncertainty is (1/9)∑_*j*=1_^9^ *m*_*j*_(5) = 0.3821.

**Step 8**

The combination of parameters (or evidences) is used to provide the strongest evidence for this medical diagnosis. By Definition 10, we can get the following results:

(33) $$\begin{array}{c} \text{Bel}\left(\left\{d_{1}\right\}\right)=m_{1}⊕m_{2}⊕m_{3}\cdots ⊕m_{9}\left(\left\{d_{1}\right\}\right)=0.0827,\\ \text{Bel}\left(\left\{d_{2}\right\}\right)=m_{1}⊕m_{2}⊕m_{3}\cdots ⊕m_{9}\left(\left\{d_{2}\right\}\right)=0.0284,\\ \text{Bel}\left(\left\{d_{3}\right\}\right)=m_{1}⊕m_{2}⊕m_{3}\cdots ⊕m_{9}\left(\left\{d_{3}\right\}\right)=0.8349,\\ \text{Bel}\left(\left\{d_{4}\right\}\right)=m_{1}⊕m_{2}⊕m_{3}\cdots ⊕m_{9}\left(\left\{d_{4}\right\}\right)=0.0471,\\ \text{Bel}\left(\Theta \right)=m_{1}⊕m_{2}⊕m_{3}\cdots ⊕m_{9}\left(\Theta \right)=0.0069. \end{array}$$

Then the final rang order is *d*_3_≻*d*_1_≻*d*_4_≻*d*_2_.

**Step 9**

According to the maximum belief measure principle, the patient is suffering from acute sinusitis *d*_3_, which is the same choice decision based on the mean potentiality approach of Example  6.2 in [22].

By Definition 7, the measure of performance of our method is the same *Υ* = 4.1726 as the mean potentiality approach's.

From above results, the belief measure of the whole uncertainty falls the originally mean value 0.3821 to 0.0751 after evidence combination. It implies that this method that combined grey relational analysis with D-S theory of evidence declines the uncertainty to a great extent and improves effectively the accuracy and reliability of medical diagnosis.

## 6. Conclusions

In this paper, we have introduced a method for fuzzy soft set in decision making by combining grey relational analysis with D-S theory of evidence and given a practical application to medical diagnosis. This method allows us to avoid the problem of selecting suitable level soft sets, reduce uncertainty caused by people's subjective cognition, and raise the decision level. Then it is more feasible and practical for dealing with applications under uncertainty. Our future work will concentrate on applications of interval-valued intuitionistic fuzzy soft sets in decision making.

## Figures and Tables

**Table 1:** Tabular representation of the soft set *(F, A).*

	*h*_1_	*h*_2_	*h*_3_	*h*_4_	*h*_5_	*h*_6_
*e*_1_	1	1	0	0	0	1
*e*_2_	1	0	0	0	0	1
*e*_3_	0	0	1	0	1	0
*e*_4_	0	0	1	1	0	1

**Table 2:** Tabular representation of the fuzzy soft set *(F, A).*

	*h*_1_	*h*_2_	*h*_3_	*h*_4_	*h*_5_	*h*_6_
*e*_1_	1	1	0.2	0.3	1	0.7
*e*_2_	1	0.1	0.3	0.2	0.1	0.9
*e*_3_	0.1	0.4	1	1	0	0.1
*e*_4_	0.1	0.3	1	1	0	1

**Table 3:** Tabular representation of the fuzzy soft set *(F, A).*

	*e*_1_	*e*_2_	*e*_3_	*e*_4_	*e*_5_
*x*_1_	0.85	0.71	0.38	0.32	0.75
*x*_2_	0.56	0.82	0.76	0.64	0.43
*x*_3_	0.84	0.51	0.82	0.53	0.47

**Table 4:** Tabular representation of the *L*((*F, A), 0.63)* with choice values.

	*e*_1_	*e*_2_	*e*_3_	*e*_4_	*e*_5_	Choice value
*x*_1_	1	1	0	0	1	3
*x*_2_	0	1	1	1	0	3
*x*_3_	1	0	1	0	0	2

**Table 5:** Tabular representation of *(F, A)* with *α*_*i*_ and *β*_*j*_ values.

	*e*_1_	*e*_2_	*e*_3_	*e*_4_	*e*_5_	*β*_*i*_
*x*_1_	0.85	0.71	0.38	0.32	0.75	0.53
*x*_2_	0.56	0.82	0.76	0.64	0.43	0.39
*x*_3_	0.84	0.51	0.82	0.53	0.47	0.37
*α*_*i*_	0.29	0.31	0.44	0.32	0.32

**Table 6:** Tabular representation of the *L*((*F, A), 0.34)* with choice values.

	*e*_1_	*e*_2_	*e*_3_	*e*_4_	*e*_5_	Choice value
*x*_1_	1	1	1	0	1	4
*x*_2_	1	1	1	1	1	5
*x*_3_	1	1	1	1	1	5

**Table 7:** Tabular representation of the soft set (*F, A).*

	*e*_1_	*e*_2_	*e*_3_	*e*_4_	*e*_5_	*e*_6_	*e*_7_
*d*_1_	0.6	0	0.6	0.9	0	0.7	0.8
*d*_2_	0.2	0	0.1	0.9	0.8	0	0
*d*_3_	0.3	0.7	0.3	0.8	0.3	0.4	0
*d*_4_	0.4	0	0.2	0.7	0.1	0.6	0.5

**Table 8:** Tabular representation of the soft set (*G, B).*

	*s*_1_	*s*_2_	*s*_3_
*d*_1_	0.6	0.8	0.4
*d*_2_	0.8	0.3	0.6
*d*_3_	0.8	0.4	0.7
*d*_4_	0.6	0.8	0.3

**Table 9:** Tabular representation of the soft set (*F*, *A*)∧(*G*, *B*).

	(*e*_1_, *s*_1_)	(*e*_1_, *s*_2_)	(*e*_1_, *s*_3_)	(*e*_2_, *s*_1_)	(*e*_2_, *s*_2_)	(*e*_2_, *s*_3_)	(*e*_4_, *s*_1_)	(*e*_4_, *s*_2_)	(*e*_4_, *s*_3_)
*d*_1_	0.6	0.6	0.4	0	0	0	0.6	0.8	0.4
*d*_2_	0.2	0.2	0.2	0	0	0	0.8	0.3	0.6
*d*_3_	0.3	0.3	0.3	0.7	0.4	0.7	0.8	0.4	0.7
*d*_4_	0.4	0.4	0.3	0	0	0	0.6	0.7	0.3
